# *ABCA1*, *ADIPOQ*, *APOE*, *FSTL4*, and *KCNQ1* Gene DNA Methylation Correlates with Lipid Profiles in Mexican Populations

**DOI:** 10.3390/biomedicines13092273

**Published:** 2025-09-16

**Authors:** Karla E. Tello-Ortega, María A. Romero-Tlalolini, Angélica Martínez-Hernández, Elizabeth Ortiz-Sánchez, Cecilia Contreras-Cubas, Humberto García-Ortiz, Francisco Barajas-Olmos, Lorena Orozco, Federico Centeno

**Affiliations:** 1 Facultad de Medicina y Cirugía, Universidad Autónoma Benito Juárez de Oaxaca, Oaxaca 68120, Mexico; ortega.biomedic@cecad-uabjo.mx; 2 Immunogenomics and Metabolic Diseases Laboratory, Instituto Nacional de Medicina Genómica, Secretaría de Salud, Tlalpan, Mexico City 14610, Mexico; amartinez@inmegen.gob.mx (A.M.-H.); ccontreras@inmegen.gob.mx (C.C.-C.); hgarcia@inmegen.gob.mx (H.G.-O.); fbarajas@inmegen.gob.mx (F.B.-O.); 3 Instituto de Salud Pública, Licenciatura en Ciencias Biomédicas, Universidad de la Sierra Sur, UNSIS, Miahuatlán de Porfirio Díaz, Oaxaca 70805, Mexico; 4 Facultad de Medicina y Cirugía, SECIHTI—Universidad Autónoma Benito Juárez de Oaxaca, Oaxaca de Juárez, Oaxaca 68120, Mexico; mdlaromerotl@secihti.mx; 5 Subdirección de Investigación Básica, Instituto Nacional de Cancerología, Mexico City 14080, Mexico; elinfkb@yahoo.com.mx

**Keywords:** DNA methylation, dyslipidemia, indigenous

## Abstract

**Background**: Dyslipidemia, a significant modifiable risk factor for cardiovascular disease (CVD), represents a major global health challenge, particularly influenced by complex genetic and environmental interactions, mainly in indigenous populations. **Methods:** In this study, DNA samples from 80 individuals belonging to various indigenous ethnic groups from northern and southern Mexico were analyzed to evaluate DNA methylation profiles and its correlation to lipid levels and other clinical parameters. Ten genes associated with metabolic changes were investigated using targeted bisulfite sequencing. **Results:** Our results revealed significant correlations between methylation in genes such as *ABCA1*, *ADIPOQ*, *APOE*, *FSTL4*, and *KCNQ1* and clinical parameters including body mass index (BMI), lipid profiles, and body fat. Of the 151 CpG sites analyzed, 16 showed statistically significant correlations. Specifically, two *ABCA1* CpGs sites correlated with BMI (*p* = 0.015) and triglycerides (*p* = 0.03); three *ADIPOQ* sites correlated with low-density lipoprotein cholesterol (LDLc) (*p* = 0.03, *p* = 0.005, *p* = 0.04, respectively); one *APOE* site correlated with BMI (*p* = 0.04), another with total cholesterol (*p* = 0.004) and triglycerides (*p* = 0.03) and two more with high-density lipoprotein cholesterol (HDLc) (*p* = 0.02 and *p* = 0.005, respectively); one *FSTL4* CpG site with body fat (*p* = 0.02), another with total cholesterol (*p* = 0.02), one more with HDLc (*p* = 0.01), and another one with triglycerides (*p* = 0.01); and two *KCNQ1* CpG sites correlated with body fat (*p* = 0.01 and *p* = 0.04, respectively). **Conclusions**: These findings show potential novel biomarkers for dyslipidemia risk. This research highlights the importance of understanding methylation changes in indigenous populations for developing personalized interventions and prevention strategies that could improve healthcare by linking epigenetic factors to CVD risk.

## 1. Introduction

Metabolic diseases are major risk factors for the development of cardiovascular diseases (CVD), such as diabetes and obesity, which are the leading cause of global morbidity and mortality [1,2].

Historically, rural and indigenous populations in Mexico were considered to have a lower risk for metabolic and cardiovascular diseases. However, recent studies showed a shifting trend, with a rising prevalence of these conditions in these groups, likely driven by changes in diet and lifestyle [3,4]. Notably indigenous communities in Mexico now exhibit some of the highest prevalence rates of metabolic syndrome worldwide, surpassing both the Mexican Mestizo population and the broader Latin American population [4].

Dyslipidemia encompasses a spectrum of abnormalities in lipoprotein synthesis, transport and metabolism, resulting in altered lipid serum levels. Notably, dyslipidemias often develop without obvious symptoms, highlighting their significant health risks [5].

The etiology of dyslipidemias is complex, arising from a confluence of genetic and environmental factors, with lifestyle playing a pivotal role as a primary risk factor. Genome-wide association studies (GWAS) have unearthed over 150 loci associated with an increased risk of dyslipidemias, including risk variants linked to other biochemical abnormalities [6]. Moreover, ethnicity is recognized as an important determinant of dyslipidemia risk [7]. While changes in lifestyle and dietary habits have contributed to the rising prevalence of cardiovascular and metabolic diseases in rural and indigenous populations, it is also recognized that these conditions are not solely influenced by external factors. This is particularly true given the greater genetic risk for metabolic diseases observed in Amerindian and Mestizo populations from Mexico. Despite this significant progress in identifying genetic mechanisms underlying these conditions, genetic markers account for only a small proportion of the variation in lipid metabolism, with some estimations as low as 15%, or even less than 7% in Mexican populations [8,9,10,11]. This discrepancy suggests that additional regulatory mechanisms, such as epigenetic modifications, play a crucial role in lipid metabolism. Epigenetics refers to heritable changes in gene expression that do not involve alterations in the DNA sequence but are influenced by environmental factors, including diet, physical activity, and exposure to toxins.

Epigenetic modifications can be dynamic, responding to lifestyle factors and potentially contributing to the high prevalence of metabolic diseases [12]. Recent research has begun to elucidate the role of epigenetic mechanisms on metabolic health, including dyslipidemia. We have previously demonstrated that alterations in DNA methylation are linked to obesity, diabetes and lipid profiles [13,14,15]. Understanding these epigenetic influences is becoming increasingly crucial in unraveling the intricate web of factors contributing to metabolic diseases, especially populations undergoing rapid epidemiological transitions. Hence, in the present study, we assess the methylation profiles of genes previously linked to metabolic alterations and examine their correlation with lipid profiles in Mexican indigenous, the population with the highest prevalence of metabolic diseases worldwide [4,16,17,18].

## 2. Materials and Methods

### 2.1. Study Population

The study included DNA samples from the peripheral blood of 80 individuals, previously genotyped and identified as Mexican Amerindians belonging to the Metabolic Analysis in an Indigenous Sample (MAIS) cohort [19]. These individuals belonged to Mayan ethnic groups from southeastern Mexico (Kaqchikel, Kanjobal, Jakalteko, Tojolabal, Chuj and Mam) (*n* = 58) as well as Tarahumara and Seri ethnic groups from northern Mexico (*n* = 22). Participants were considered Amerindian if they identified themselves as indigenous, had both parents and grandparents born in the same community, and spoke the native language. Ancestry was confirmed in a random sample using the 6.0 SNP array (Affymetrix Inc., Sunnyvale, CA, USA). They had an average Amerindian ancestry of 95% (standard deviation 5.7%) [4,19]. Anthropometric, demographic, personal and family medical history, and lifestyle data were collected through participant interviews conducted by trained staff from INMEGEN, using a standardized questionnaire. Body fat percentage was evaluated by bioelectrical impedance with a Body Composition Monitor HBF-500 INT (Omron Healthcare Mexico S.A. de C.V., Mexico City, Mexico).

A peripheral blood sample was collected after a fast of at least 8 hours. Biochemical analyses, including fasting glucose, total cholesterol (TC), high density lipoprotein cholesterol (HDL-C), and triglycerides (TGs), were carried out using a Cholestech LDX Analyzer (Abbott, Abbott Park, IL, USA).

This research was conducted with the approval and collaboration of the indigenous leaders. All participants provided written informed consent, and authorities or community leaders participated as translators as necessary. The samples were collected between 2011 and 2015.

This study was conducted in accordance with the Declaration of Helsinki and was approved by the Research, clinical trials, and Biosafety Committees of the Instituto Nacional de Medicina Genómica (INMEGEN) in Mexico City (protocol number 31/2011/I).

### 2.2. Gene Selection and Primer Design

A panel of 10 genes was selected based on our previous research [14,15], that documented genes exhibiting altered methylation profiles related to metabolic parameters. The selected genes included *ABCA1*, *ADIPOQ*, *AIM2*, *APOE*, *FSTL4*, *FTO*, *GNAS*, *KCNQ1*, *LCLAT1* and *ZNF714*. Specific primers for bisulfite-converted DNA of these selected genes were designed with MethPrimer [20]. Primer sequences are listed in Supplementary Table S1. Adapter sequences were added to primers and primer specificity and efficiency were validated by conventional PCR and analyzed using an Agilent 2100 bioanalyzer (Agilent Technologies Inc., Santa Clara, CA, USA).

### 2.3. Methylation Analysis

Methylation analysis was performed with targeted bisulfite next generation sequencing. Bisulfite conversion of genomic DNA was carried out using the EZ DNA Methylation Kit (Zymo Research, Irvin, CA, USA) following the manufacturer’s instructions. This process converts unmethylated cytosines to uracil, while methylated cytosines remain unchanged, enabling the subsequent analysis of DNA methylation patterns.

PCR amplified bisulfite-converted DNA was used as input for targeted bisulfite sequencing library preparation using the Illumina TruSeq Methyl-Seq Kit (Illumina, Inc., San Diego, CA, USA). Libraries were purified, quantified, and pooled in equimolar amounts. The pooled library was then denatured and clustered onto a MiSeq flow cell. Sequencing was performed on a MiSeq sequencer (Illumina, Inc., San Diego, CA, USA) using the Reagent Kit v2 (Illumina, Inc., San Diego, CA, USA) to generate paired-end reads of 250 bp.

### 2.4. Sequencing Data and Bioinformatic Analyses

Raw sequencing reads were initially assessed for quality control using FastQC. Low-quality reads were trimmed using Trimmomatic to remove adapter sequences and bases with low Phred quality scores [21]. The cleaned reads were then aligned to the human reference genome (hg38) using Bismark, accounting for the specific mapping challenges associated with bisulfite-converted DNA. Methylation calls were generated using the Bismark methylation extractor. Finally, the methylation data were analyzed using the EPIC-TABSAT pipeline (https://tabsat.ait.ac.at/, accessed on 20 May 2022).

A total of 184 CpG sites were assessed, of which 151 reached a depth of over 200 reads and were considered for further analysis. Data analysis was performed using the free software environment R 3.6.2, along with the graphical interfaces of the integrated development environment R Studio 8.14 and R Commander 2.5-x.

### 2.5. Statistical Analyses

We analyzed regional differences in DNA methylation between communities Mayan (South) and Tarahumara and Seri (North). Given the limited sample size per community, we chose to perform the regional analysis by comparing the north and south. Additionally, we explored the relationship between methylation profiles and variables such as ethnicity, sex, age, BMI, body fat and serum lipid levels. The variables passed the normality test, and therefore, Pearson’s correlations were performed between methylation levels and clinical variables. Correlation analysis was conducted to examine the percentage of methylation at each CpG site in relation to the quantitative measurements of each variable.

## 3. Results

### 3.1. Clinical Data of Participants

Samples were drawn from the MAIS cohort previously described [19]. For this analysis, a total of 80 patients from various ethnic groups across northern and southern Mexico were selected. A summary of the clinical parameters for these patients is provided in Table 1. The participants had an average age of 42.4 ± 4.3 years. Significant differences were found between the northern and southern region in body fat percentage and lipid profiles, as well as in the proportion of individuals presenting altered values of total cholesterol (50% north, 21% south), HDLc (41% north, 66% south) triglycerides (59% north, 76% south), and LDLc (27% north, 19% south), regardless of sex (Supplementary Figure S1).

Notably, the incidence of dyslipidemia in this sample is remarkably high, especially regarding hypertriglyceridemia and hypoalphalipoproteinemia, mirroring the elevated levels we previously observed [4].

### 3.2. DNA Methylation and Correlation with Clinical Data

We analyzed the methylation profiles of specific sites within several genes previously associated with metabolic processes using targeted bisulfite next-generation sequencing. The following 10 genes and CpG sites were examined: *ABCA1* (5 sites), *ADIPOQ* (12 sites), *AIM2* (9 sites), *APOE* (14 sites), *FSTL4* (7 sites), *FTO* (13 sites), *GNAS* (15 sites), *KCNQ1* (12 sites), *LCLAT1* (36 sites) and *ZNF714* (28 sites). In total, we assessed the methylation status of 151 CpG sites. In our analysis, no clustering by ethnic group or geographic region of Mexico was observed across all the gene regions examined (not shown), suggesting that the methylation patterns studied are consistent across individuals regardless of their ethnic or regional background.

We then analyzed the correlation between the methylation and lipid profiles, as well as clinical parameters such as BMI and body fat percentage.

**Figure 1:**
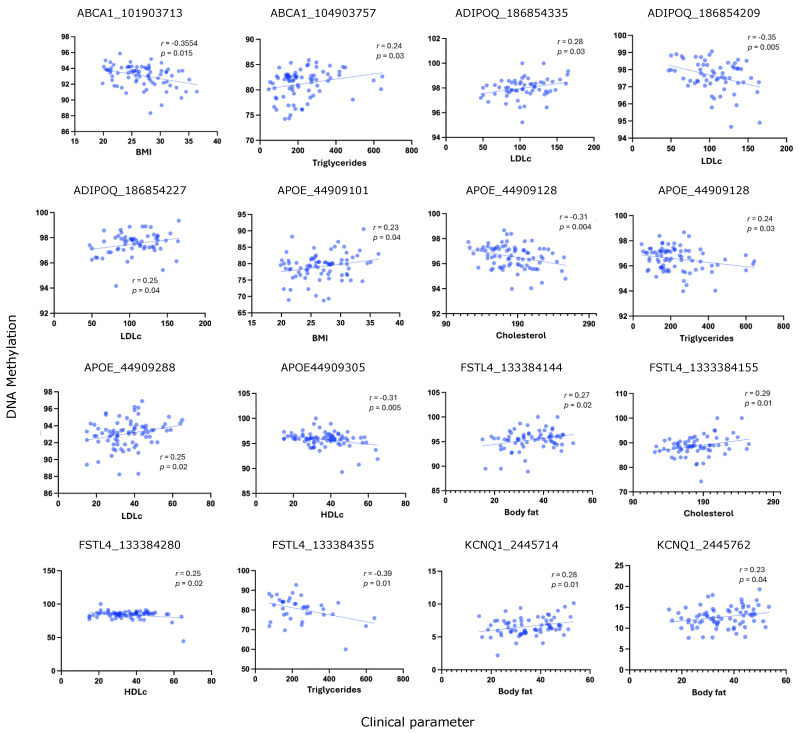
Correlation between clinical parameters and DNA methylation in Mexican populations. Plots show correlation between clinical parameters—Body Mass Index (BMI), body fat percentage, total cholesterol, low-density lipoprotein (LDLc), high-density lipoprotein (HDLc), and triglycerides—and DNA methylation levels at CpG sites with significant correlation. Each plot represents a CpG–parameter pair, indicated by the name of the gene and CpG genome location. Each blue dot represents the values for each individual patient.

A total of 16 sites across five genes (*ABCA1*, *ADIPOQ*, *APOE*, *FSTL4* and *KCNQ1*) showed a statistically significant correlation at a 95% confidence interval (*p* < 0.05). Seven of these 16 sites exhibited a negative correlation, while nine displayed a positive correlation (Table 2, Figure 1). No significant correlations were found between DNA methylation and clinical parameters within the genes *AIM2*, *FAM134C*, *GNAS*, *LCLAT1*, and *ZNF714*.

## 4. Discussion

In recent years, the increasing prevalence of metabolic diseases has underscored the urgent need to comprehensively identify all factors contributing to their pathogenesis. In previous research, we established correlations between the methylation levels at specific gene sites and clinical parameters related to obesity and type 2 diabetes.

In this study, we aimed to research the relationship between DNA methylation profiles of specific genes involved in lipid metabolism and metabolic clinical parameters in indigenous Mexican populations. The variability in methylation profiles can be attributed to a combination of genetic, environmental, and other epigenetic factors. Understanding these differences is crucial for comprehending disease susceptibility and health disparities across populations. In this study, we analyzed methylation profiles in indigenous communities from northern and southeastern Mexico. Although the set of analyzed CpG sites did not allow discrimination by geographic region or ethnicity, this does not rule out the possibility that other CpG sites could show regional or ethnic clustering. This finding indicates that the specific gene regions studied may be influenced by common environmental factors or intrinsic biological processes that transcend regional and ethnic differences. On the other hand, our study reveals significant correlations between DNA methylation at specific CpG sites in several genes and various clinical parameters, providing insights into potential epigenetic mechanisms affecting metabolic health.

For the *ABCA1* gene, we observed that methylation of two CpG sites correlated with BMI and triglyceride levels. The *ABCA1* gene plays a crucial role in lipid efflux and HDL cholesterol biogenesis. Prior studies have shown that *ABCA1* expression is influenced by epigenetic modifications, including DNA methylation [22]. For example, An et al. (2021) reported that *ABCA1* methylation is linked to obesity and cardiovascular disease risk in various populations [23]. Our findings suggest that methylation could be a potential mechanism through which *ABCA1* influences body weight regulation. Moreover, our results align with research indicating that altered methylation can affect lipid metabolism, contributing to dyslipidemia and cardiovascular disease [24,25].

The *ADIPOQ* gene, which exhibited correlations at multiple CpG sites with LDL cholesterol levels, encodes adiponectin, a hormone involved in glucose regulation and fatty acid oxidation. *ADIPOQ* methylation has been associated with insulin resistance and other metabolic parameters [26,27]. Our findings further strengthen the evidence for the central role of adiponectin in regulating lipid metabolism and its critical connection to CVD risk.

The central role of *APOE* in lipid metabolism and cardiovascular health is well-documented, as this gene plays a pivotal role in lipid transport, clearance, and homeostasis. *APOE* variants have been widely linked to plasma cholesterol and triglyceride levels, highlighting its importance in determining susceptibility to cardiovascular diseases. In our study, several CpG sites within the *APOE* gene correlated with BMI, cholesterol and triglycerides. These results are consistent with studies that have identified differential methylation in *APOE* as a marker for lipid levels and cardiovascular risk [28]. Furthermore, methylation at two CpG sites within this gene were inversely related to HDL cholesterol, supporting findings by Domingo-Relloso et al. (2022), who noted similar associations in populations exposed to environmental pollutants [29].

Other important correlated CpG sites were found within the *FSTL4* gene, whose impact of its methylation in humans remains largely unknown. In this study we found significant correlations with several clinical parameters, such as body fat, total cholesterol, HDL cholesterol, and triglycerides. While information on the function of this gene is scarce, it is known to participate in heart development, and its underexpression has been linked to cardiac dysfunction in mice [30,31]. In adults, the highest expression of this gene is found in bladder and nervous tissue. Given our results, future studies integrating methylation and expression analyses in metabolically active tissues, as well as functional assays in cellular and animal models, will be crucial to determine whether epigenetic regulation of FSTL4 contributes to metabolic and cardiovascular phenotypes.

On the other hand, genetic variants in *KCNQ1*, primarily known for its role in cardiac electrical activity, have been associated with obesity, type 2 diabetes (T2D), and stroke [32,33,34]. Furthermore, Hu et al. (2021) demonstrated a complex interplay between genetic variants and methylation of *KCNQ1* in risk of developing T2D [35]. Our findings show a correlation between two *KCNQ1* sites and body fat percentage, further support the emerging role of this gene in metabolic diseases. This aligns with our previous research, which suggested a link between *KCNQ1* methylation and obesity and metabolic health [12,13,14].

Interestingly, contrary to those observed previously by our research in adipose tissue of patients with obesity and other metabolic diseases, in blood samples we did not observe significant correlations between clinical parameters and CpG methylation within the *AIM2*, *FAM134C*, *GNAS*, *LCLAT1*, and *ZNF714* [12,13,14]. This may be attributed to tissue-specific differences in DNA methylation patterns, since adipose tissue directly reflects metabolic processes, while whole blood may not fully capture the relevant epigenetic modifications to these genes and their involvement in metabolic regulation. Although this could be considered a limitation of the study, it also offers a valuable opportunity to identify genes whose alterations in peripheral blood may reflect changes in the target tissues of metabolic diseases. The added convenience of minimally invasive sample collection facilitates the identification of potential biomarkers for metabolic disease in broader clinical and epidemiological studies, and highlights their potential application in personalized medicine, where biomarker-based approaches can improve early detection, risk stratification, and tailored interventions.

To our knowledge, this is the first study to analyze the correlation between DNA methylation and lipid profiles in Mexican indigenous communities. Future research should integrate as much information as possible, including both genetic and epigenetic data, to gain a deeper understanding of the molecular mechanisms of metabolic diseases in these populations or in others with a high incidence of these diseases. Additionally, future studies should include non-indigenous groups for comparison to better understand the unique epigenetic factors contributing to health disparities in these communities.

Despite the relevance of these findings, some limitations should be acknowledged. The relatively small sample size (*n* = 80) may limit the statistical power and generalizability of our results. Although the observed correlations were significant but modest in strength, they are consistent with those reported in previous studies of DNA methylation and metabolic traits [16], reinforcing the biological plausibility of our findings. Nevertheless, future studies are needed to further explore the function of these genes and their potential role in metabolic diseases, especially to determine the effect of methylation on gene expression in the target tissue related to these diseases.

## 5. Conclusions

This study provides novel insights into the interplay between epigenetics and lipid metabolism in indigenous Mexican populations. Our findings demonstrate significant correlations between DNA methylation levels at specific CpG sites within key genes (*ABCA1*, *ADIPOQ*, *APOE*, *FSTL4*, and *KCNQ1*) and clinically relevant parameters, such as BMI, total cholesterol, HDL cholesterol, triglycerides, and body fat percentage. These findings underscore the potential importance of epigenetic mechanisms in the development of dyslipidemias within these populations. Overall, this paper highlights the consistency in the links between these methylation patterns with metabolic health across geographic regions and ethnicities, suggesting that nationwide public health strategies may be beneficial for addressing metabolic disorders in Mexican populations.

This study suggests that DNA methylation may serve as a valuable biomarker for assessing individual risk to dyslipidemia within Mexican communities.

Further research exploring the functional implication of these methylation changes and their potential as biomarkers, could be needed to implement novel therapeutic approaches to a personalized medicine targeted to metabolic diseases.

## Figures and Tables

**Table 1:** Clinical parameters of participant.

	Region		
	North	South	
Age (years)	42.9 ± 4.9	42.1 ± 4.2	
Triglycerides (mg/dL)	212.2 ± 162.8	232.5 ± 115.7	*p* = 0.59
Triglycerides ≥ 150 mg/dL)	59%	76%	
Total cholesterol (mg/dL)	198.7 ± 33.2	176.9 ± 28.1	***p* = 0.004**
Total cholesterol ≥ 200 mg/dL	50%	21%	
LDLc (mg/dL)	122.3 ± 21.9	100.6 ± 29.3	***p* = 0.001**
LDLc ≥ 130 mg/dL	27%	19%	
HDLc (mg/dL)	42.8 ± 13.3	33.8 ± 9.7	***p* = 0.007**
HDL < 40 mg/dL	41%	66%	
BMI (kg/m^2^)	27.0 ± 4.4	26.7 ± 3.8	*p* = 0.775
Overweight	41%	41%	
Obesity	27%	24%	
Body fat (%)	40.1 ± 8.9	34.6 ± 9.6	***p* = 0.021**

*p* values in bold are < 0.05.

**Table 2:** Correlation between methylation and clinical parameters. *p* values for the correlation between DNA methylation and clinical parameters.

Gene	CpG Location	Gene Region	BMI	BF	TC	TG	LDLc	HDLc
*ABCA1*	Chr9: 104903713	5′UTR	**0.015**	0.2	0.7	0.2	0.4	0.1
	Chr9:104903757	5′UTR	0.4	0.5	0.1	**0.03**	0.8	0.9
*ADIPOQ*	Chr3: 186854335	CDS	0.3	0.9	0.5	0.8	**0.03**	0.9
	Chr3: 186854209	CDS	0.1	0.7	0.1	0.7	**0.005**	0.4
	Chr3: 186854227	CDS	0.7	0.2	0.3	0.3	**0.04**	0.2
*APOE*	Chr19: 44909101	CDS	**0.04**	0.7	0.9	0.2	0.1	0.7
	Chr19: 44909128	CDS	0.8	0.3	**0.004**	**0.03**	0.3	0.3
	Chr19: 44909288	3′UTR	0.5	0.5	0.6	0.4	0.2	**0.02**
	Chr19: 44909305	3′UTR	0.2	0.5	0.9	0.7	0.3	**0.005**
*FSTL4*	Chr5: 133384144	Intron	0.7	**0.02**	0.6	0.9	0.9	0.8
	Chr5: 133384155	Intron	0.7	0.8	**0.01**	0.9	0.3	0.1
	Chr5: 133384280	Intron	0.2	0.9	0.2	0.8	0.2	**0.01**
	Chr5: 133384355	Intron	0.4	0.7	0.7	**0.01**	0.6	0.4
*KCNQ1*	Chr11: 2445714	Intron	0.9	**0.01**	0.1	0.5	0.4	0.7
	Chr11: 2445762	Intron	0.5	**0.04**	0.9	0.4	0.3	0.8

In bold *p* values < 0.05. Body mass index (BMI), body fat percentage (BF), total cholesterol (TC), triglycerides (TG), low-density lipoprotein cholesterol (LDLc), high-density lipoprotein cholesterol (HDLc).

## Data Availability

The data presented in this study are available on request from the corresponding author.

## Supplementary Materials

The following supporting information can be downloaded at: https://www.mdpi.com/article/10.3390/biomedicines13092273/s1, Figure S1: Regional differences in clinical parameters. Table S1: Primers for PCR amplification.
