# Supplementary figures and images for: ABCA1, ADIPOQ, APOE, FSTL4, and KCNQ1 Gene DNA Methylation Correlates with Lipid Profiles in Mexican Populations
Source: Biomedicines. 2025 Sep 16;13(9):2273. doi: 10.3390/biomedicines13092273 (PMC12467348; doi:10.3390/biomedicines13092273)

## Supplementary figure S1. Regional distribution of clinical parameters

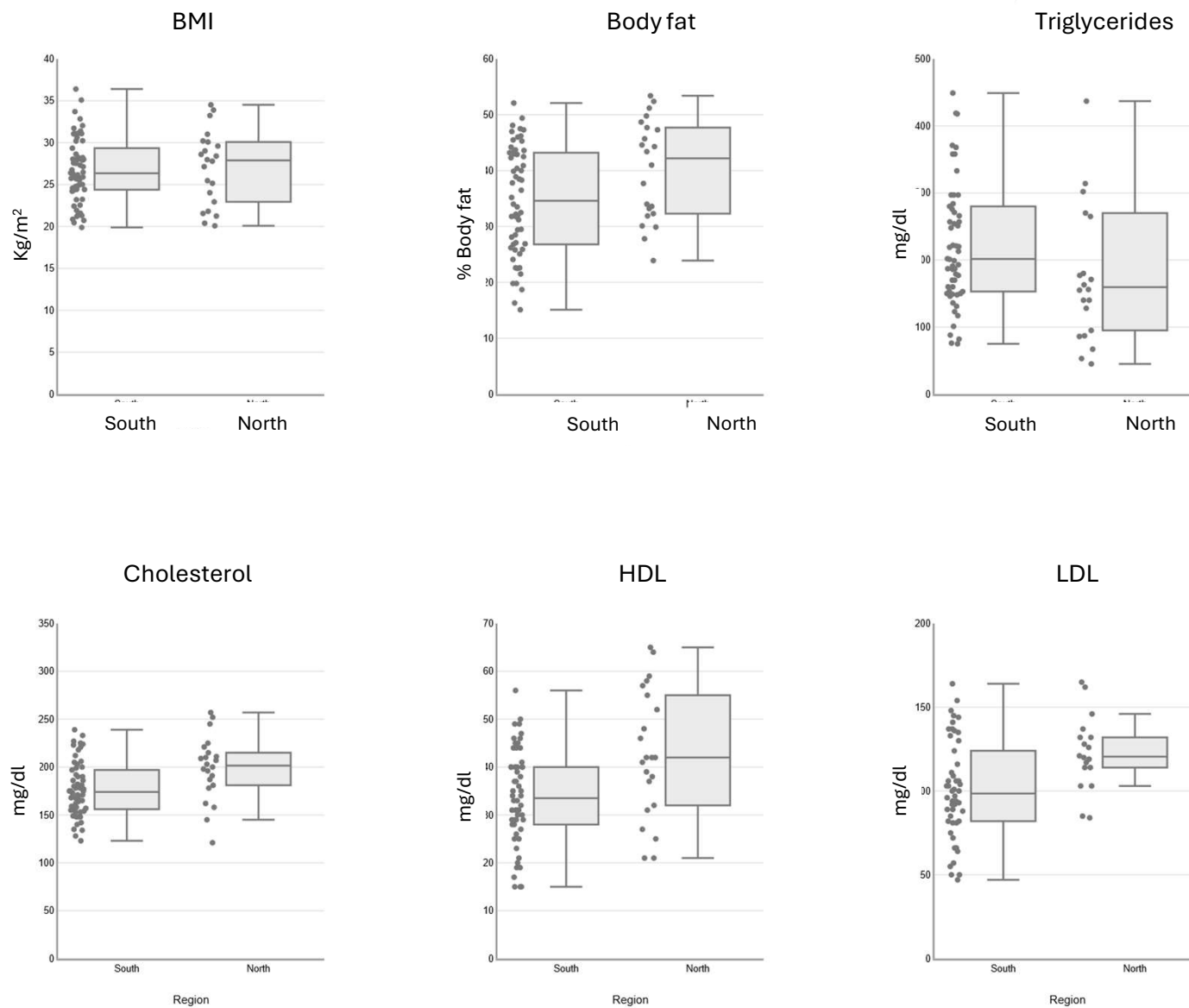

Supplement: Supplementary file 1 [file biomedicines-13-02273-s001.zip › Fig_S1.pdf]
